# Alternative initiations of three-monthly and six-monthly paliperidone palmitate in a psychiatric hospitalization unit. About two clinical cases.

**DOI:** 10.1192/j.eurpsy.2023.2318

**Published:** 2023-07-19

**Authors:** V. Juárez Calvo, C. Rodríguez Villarino, M. Presa García

**Affiliations:** Servicio de Psiquiatría y Salud Mental, Hospital Central de la Defensa Gómez Ulla Centro Sanitario de Vida y Esperanza, Madrid, Spain

## Abstract

**Introduction:**

Long acting injectable antipsychotics (LAIA) have been an important therapeutic advance. Due to poor adherence, in some patients the only way to ensure the continuity of outpatient care is to anticipate the use of a 3-monthly or 6-monthly LAIA. There is already experience of alternative initiations with 6-monthly and 3-monthly paliperidone palmitate with with an excellent tolerability and efficacy profile, following the same alternative initiation regimen used in these clinical cases.

**Objectives:**

The aim of the present study is to describe the alternative initiations with 3-monthly paliperidone palmitate (PP3) and 6- monthly paliperidone palmitate (PP6) carried out at the brief hospitalization unit of psychiatry of Hospital Central de la Defensa Gómez Ulla, Centro Sanitario de Vida y de Esperanza

**Methods:**

We report two clinical cases. With regard to two patients diagnosed with schizophrenia, with poor adherence to treatment previously prescribed with monthly LAIA, refusal to take oral medication and with serious behavioral changes due to psychotic relapses, the process of psychopathological stabilization is described with an alternative initiation of PP6 and PP3.

**Results:**

Both patients had a diagnosis of schizophrenia, being men of 32 and 28 years of age admitted to the brief hospitalization unit of psychiatry, who presented important behavioral alterations due to sensory-perceptive alterations and the delusional ideas they presented. Initially, psychopathological stability was achieved, remission of all symptoms, with oral paliperidone 12 mg in both patients. Subsequently, the alternative initiation scheme was followed, consisting of administering on the same day (day 1) 150 mg of 1-monthly paliperidone palmitate (PP1) together with 1000 mg of 6-monthly paliperidone palmitate in one of the patients, and in the other patient, on the same day (day 1) 150 mg of 1-monthly paliperidone palmitate and 525 mg of 3-monthly paliperidone palmitate. Both patients maintained psychopathological stability, allowing early hospital discharge and no decompensation occurring during the following 6 months of follow-up.

**Conclusions:**

There is an important group of patients with severe mental disorder that could benefit from an alternative initiation with 3/6-monthly paliperidone palmitate, rather than the standard initiation with monthly paliperidone palmitate. We present two patients who have greatly benefited from an alternative initiation, with the structure of PP1 150 mg + PP3 525 mg (both administered on day 1) and PP1 150 mg + PP6 1000 mg (both administered on day 1). The use of these alternative starts with PP3 and PP6 may be an important clinical tool for less adherent patients. More studies are needed to confirm these results.

**Disclosure of Interest:**

None Declared

